# Effect of Diet Modification on Salivary Parameters and Oratest in High-caries-risk Individuals

**DOI:** 10.5005/jp-journals-10005-1480

**Published:** 2017-02-01

**Authors:** Sonal Jain, Kalpana Bansal, Mohita Marwaha, Nidhi Sehrawat, Shanal Singla

**Affiliations:** 1 Senior Lecturer, Jubesta Hospital, Raipur, Chhattisgarh, India; 2 Assistant Professor, Department of Pedodontics and Preventive Dentistry, All India Institute of Medical Sciences, New Delhi, India; 3 Reader, Department of Pedodontics, SGT Dental College Hospital & Research Institute, Gurugram, Haryana, India; 4 Private Practitioner, Department of Pedodontics, Toothmates Dental Clinic, New Delhi, India; 5 Private Practitioner, Department of Pedodontics, Toothmates Dental Clinic, New Delhi, India

**Keywords:** Dental caries, Diet counseling, Oratest, Saliva.

## Abstract

**Aim:**

This study was aimed to assess the salivary parameters and caries activity test (Oratest) in high-caries-risk individuals and effect of diet modification and parental education on salivary parameters and Oratest.

**Materials and methods:**

Forty-five children aged between 5 and 8 years and decayed, extracted due to caries, filled teeth (deft)/decayed, missing, filled teeth (DMFT) scores >5 were selected and evaluated for salivary parameters, such as hydration status of oral mucosa, resting pH, unstimulated salivary flow rate (USFR) and stimulated salivary flow rate (SSFR), buffering capacity of stimulated saliva (BCSS) before and after diet counseling using GC India Saliva Check Kit. Oratest was performed to assess the caries activity. Children’s parents were asked to record 5 to 7 days diet chart. Diet charts were collected and based on the evaluation of specific diet charts, diet counseling was provided. After 6 weeks, salivary parameters and Oratest were reevaluated. Baseline and postdiet counseling salivary parameters were subjected to statistical analysis using Student’s t-test (paired) and Wilcoxon signed rank test.

**Results:**

From baseline to 6 weeks, USFR and SSFR were increased which were statistically significant. Buffering capacity and resting pH increased slightly but was not statistically significant. The reading of Oratest increased significantly, indicating a decreased caries activity in individuals.

**Conclusion:**

It can be concluded that diet counseling, parental education, and regular motivation can positively alter salivary parameters, such as USFR and SSFR.

**How to cite this article:** Jain S, Bansal K, Marwaha M, Sehrawat N, Singla S. Effect of Diet Modification on Salivary Parameters and Oratest in High-caries-risk Individuals. Int J Clin Pediatr Dent 2018;11(1):34-39.

## INTRODUCTION

The association between sugar intake and dental caries is widely accepted, but the effect of other ingredients or fiber content of the diet has been scarcely studied. Diets requiring mechanical chewing are known to be beneficial for dental health because of the resulting increase in salivary flow rate. In general, when the salivary flow rate increases, the buffering capacity increases.^1^ Bioactive compounds found in some foods, e.g., bioactive casein phosphopep-tides in dairy products, tea, and cranberry-derived poly-phenols may protect teeth, e.g., from erosion^2,3^ and caries.^4,5^

The etiology and pathogenesis of dental caries are known to be multifactorial involving internal defense factors, such as saliva, tooth surface morphology, general health, nutritional and hormonal status, and a number of external factors—e.g., diet, the microbial flora colonizing the teeth, oral hygiene, and fluoride availability.^6^ Alterations in the physicochemical properties of saliva, such as decreased salivary flow rate, pH, buffering capacity, play a major role in the development of caries.^7^

A simple caries activity test, Oratest, was developed by Rosenberg et al^8^ and Tal and Rosenberg^9^ based on the rate of oxygen depletion in expectorated milk samples. It is a simple, economical, noninvasive, and less time-consuming test for estimating the oral microbial level.^10^

Very few studies have been conducted to determine the effect of diet modification and parental education on salivary parameters.^1,11,12^ This study was planned with the following aims and objectives: Firstly, to evaluate the baseline salivary parameters—hydration status of oral mucosa, USFR and SSFR, resting pH, and BCSS in subjects with high caries index (DMFT/deft>5). Secondly, the aim was to evaluate the effect of diet modification on salivary parameters in subjects with high caries index. Thirdly, the objective was to compare the susceptibility to caries using simple chair-side caries activity test, Oratest, before and after diet modification.

## MATERIALS AND METHODS

This study was conducted in the Department of Paedo-dontics and Preventive Dentistry, SGT Dental College, Gurgaon. Ethical clearance was obtained from the Ethical Committee of the Institute. A total of 45 children aged 5 to 8 years were randomly selected in the study from the Department of Paedodontics and Preventive Dentistry at SGT Dental College. Following were inclusion and exclusion criteria:

### Inclusion Criteria

- Children in the age group of 5 to 8 years
- Free from any systemic or local diseases which can affect salivary secretions (such as submandibular duct canaliculi, asthma, and diabetes mellitus)
- Caries-active children having DMFT/deft score >5

### Exclusion Criteria

- History of current or recent (at least for the past 1 month) antibiotic usage
- Children who were severely ill and having difficulty in opening the mouth
- Abscess, draining sinus, cellulitis, or other conditions requiring emergency dental treatment
- Children with any orthodontic appliances or any removable/fixed prostheses
- Children with any oral habit (e.g., mouth breathing)

### Armamentarium

**Fig. 1:**
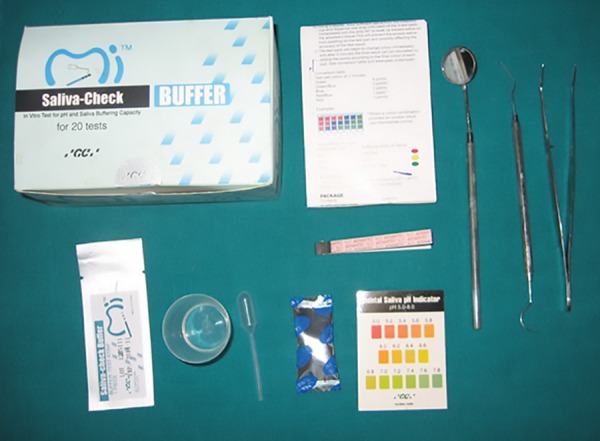
Armamentarium for evaluation of salivary parameters

**Fig. 2:**
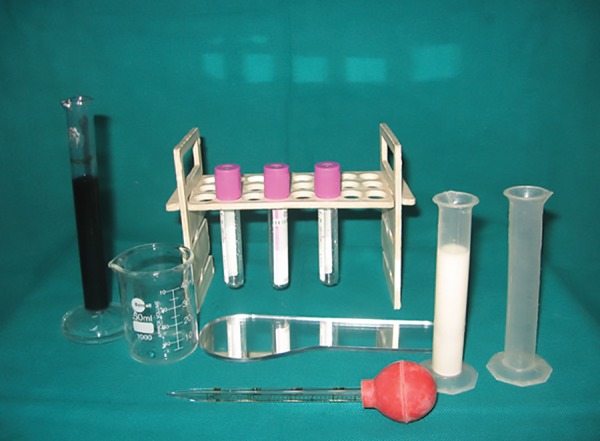
Armamentarium for Oratest

Probe, Mirror, Tweezer, GC India Saliva Check kit (graduated containers, plastic pipettes, pH test paper, buffer test strips, and paraffin tablets) (Fig. 1). For Oratest, sterilized cow milk, 0.1% aqueous solution of methylene blue, beakers, test tubes, 5 mL disposable syringes, mirror, and test tube stand (Fig. 2).

## PROCEDURE

A total of 45 children were selected for the study who fulfilled the inclusion criteria and whose parents consented to participate in the study. Dental caries scores were assessed using modified DMFT and deft index under dental light using a plane mouth mirror and blunt probe. Prior to salivary collection, the patient was instructed to refrain from eating or drinking anything for at least one hour. Salivary parameters, such as hydration status of oral mucosa, resting pH, USFR, SSFR, and BCSS were evaluated using GC India Saliva Check Kit.

The hydration status of oral mucosa was recorded as time taken for saliva droplets to be formed on the lower lip. The USFR and SSFR were recorded by collecting saliva in a graduated container. The BCSS was recorded by comparing the color change of buffering strips with the standard color chart as provided with the GC India saliva check kit.

**Fig. 3:**
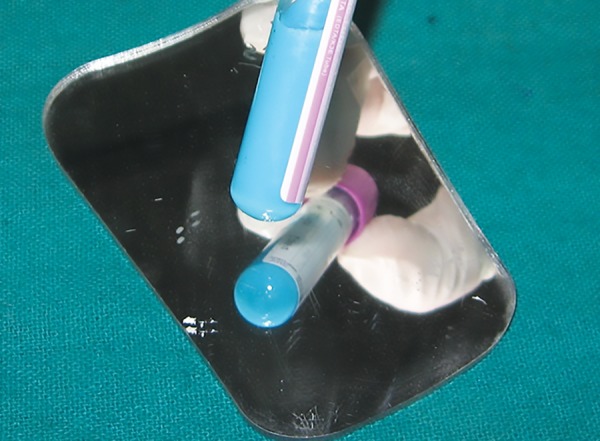
Oratest—the bottom of the test tubes, prior to and following the blue-to-white color change

**Fig. 4:**
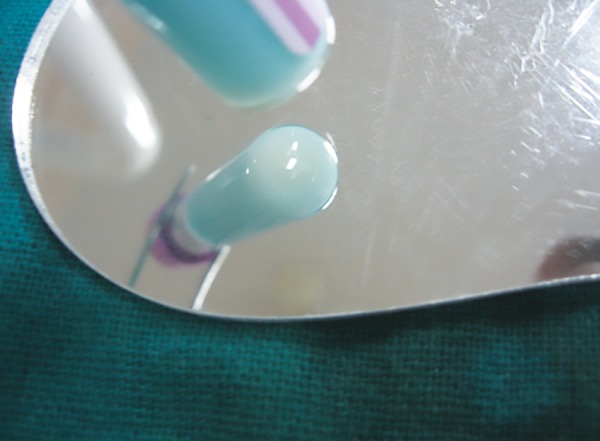
Oratest—the bottom of the test tubes, prior to and following the blue-to-white color change after 10 minutes

Caries susceptibility was evaluated using Oratest. For this, the patient was asked to rinse the mouth with 10 mL of sterile milk for 30 seconds and was instructed to expectorate in a beaker, 3 mL of which was mixed with 0.1 mL of 0.1% methylene blue dye in a glass test tube. Time was noted and the test tube was held still in a test tube stand. The bottom of the test tube was observed for color change using a mirror after every 10 minutes (Fig. 3). When a white precipitate was formed of at least 6 mm diameter, time was noted again (Fig. 4).^8,9^ So, the total time taken for the precipitate to form was recorded in minutes.

### Diet Counseling

Following this, the parent and patient were instructed to record 5 to 7 days diet diary. Parents were asked to keep a running daily record of meals and between-meal snacks for 5 to 7 consecutive days, including a weekend day or holiday. The diet diary was evaluated thoroughly after 5 to 7 days. Dietary instructions were given to add foods which require more chewing like raw fruits, vegetables, dairy products, and high-fiber foods in their diet. Also, they were encouraged to minimize the intake of sugary and processed food in their diet. These patients were recalled every week for diet counseling and motivation for a duration of 6 weeks. After 6 weeks, salivary and caries susceptibility tests (Oratest) were repeated. Thus, a total of two salivary samples were taken of each individual. Baseline and postdiet counseling salivary parameters and Oratest were analyzed and compared using Statistical Package for the Social Sciences version 17. Student’s paired t-test and Wilcoxon signed rank test were performed.

## RESULTS

The hydration status of oral mucosa—improvement in the hydration status of oral mucosa—was seen in only two individuals postdiet counseling. The p-value was 0.157, which was statistically nonsignificant (Table 1).

USFR: The mean USFR at baseline was 0.309 mL/ minute, which increased to 0.321 mL/minute postdiet counseling, i.e., after 6 weeks. The p-value was 0.04, which was statistically significant (Table 2).

SSFR: The mean SSFR at baseline was 0.487 mL/ minute, which increased to 0.556 mL/minute postdiet counseling, i.e., after 6 weeks. The p-value was 0.001, which was statistically significant (Table 2).

Resting pH: The mean resting pH at baseline was 7.049, which increased to 7.084 postdiet counseling, i.e., after 6 weeks. The p-value was 0.29, which was statistically not significant (Table 2).

**Table 1:** **Table 1:** Comparison of mean baseline and postdiet counseling values of hydration status of oral mucosa

*Hydration status of oral mucosa*		*N*		*Negative ranks (after < before)*		*Positive ranks (after > before)*		*Ties*		*p-value*	
After-before		45		2		0		43		0.157	
										NS	

NS: Not significant

**Table 2:** **Table 2:** Statistical comparison between mean baseline and postdiet counseling values of USFR, SSFR, resting pH, BCSS, and reading of Oratest

*Parameter*		*n*		*Before (mean ± SD)*		*After (mean ± SD)*		*p-value*	
USFR (mL/min)		45		0.309 ± 0.106		0.321 ± 0.096		0.04*	
SSFR (mL/min)		45		0.487 ± 0.171		0.556 ± 0.169		0.001**	
Resting pH		45		7.049 ± 0.359		7.084 ± 0.297		0.29	
BCSS		45		9.578 ± 0.783		9.711 ± 0.626		0.057	
Reading of		45		82.778 ± 17.6		99.667 ± 16.7		0.000**	
Oratest									

*Significant; **Highly significant

Buffering capacity: The mean BCSS at baseline was 9.578, which increased to 9.711 postdiet counseling, i.e., after 6 weeks. The p-value was 0.057, which was statistically not significant (Table 2).

Oratest: The mean readings of Oratest at baseline was 82.778 minutes which increased to 99.667 minutes postdiet counseling, i.e., after 6 weeks. The p-value was 0.000, which was statistically significant (Table 2).

## DISCUSSION

Many factors in addition to sugar affect the caries process, including the form of food or fluid, the duration of exposure, nutrient composition, sequence of eating, salivary flow, presence of buffers, and oral hygiene.^13^

### Degree of Hydration

Dawes reported that when body water content was reduced by 8%, salivary flow decreased virtually to zero. In contrast, hyperhydration causes an increased salivary flow rate.^14^ If droplets cannot be seen after 60 seconds, the resting salivary flow is below the normal range, and this should be investigated.^15^

In the present study, 8 out of 45 participants (17.8%) had baseline hydration status low, as droplets appeared after 60 seconds. Rest 82.2% subjects had normal hydration status. This finding is in correlation with the study conducted by Hedge et al,^16^ who found that there is no association between caries activity and hydration status of oral mucosa. Gopinath and Arzreanne^17^ reported results of hydration status in 20 high-caries-risk individuals in which, in 90% of subjects, droplets appeared after 30 seconds. This difference was due to the difference in the time period in both the studies. In the present study, 6 out of 45 subjects had low hydration status after 6 weeks, i.e., there was improvement in the hydration status in only two individuals postdiet counseling, which was not statistically significant.

### Unstimulated and Stimulated Salivary Flow Rates

Saliva flow is termed “unstimulated” when no exogenous or pharmacological stimulation is used and is termed “stimulated” when secretion is promoted by mechanical or gustatory stimuli or by pharmacological agents.^14^ A healthy person’s mean daily saliva production ranges from 1 to 1.5 L.^18^ In adults, the normal total stimulated salivary flow ranges from 1 to 3 mL/minute, low ranges from 0.7 to 1.0 mL/minute, while hyposalivation is characterized by a salivary flow of less than 0.7 mL/ minute. The normal unstimulated salivary flow ranges from 0.25 to 0.35 mL/minute, low ranges from 0.1 to 0.25 mL/minute, while hyposalivation is characterized by an salivary flow of less than 0.1 mL/minute.^19^

In the present study, the mean USFR prior to diet counseling was 0.309 mL/minute, which was increased to 0.321 mL/minute after diet counseling. This finding is in correlation with several studies^20-23^ which found mean USFR as 0.39, 0.32, 0.31, 0.29 mL/minute respectively in children. Results of the present study were in contrast to the study conducted by Preethi et al^7^ who stated that there was no correlation between USFR and caries activity. Similar results were given by Tulunoglu et al.^22^ Differences in values may be due to different weather conditions, as subjects may get dehydrated in summer, resulting in a reduced flow rate.^14^

In the present study, the mean SSFR prior to diet counseling was 0.49 mL/minute (0.3-1.2 mL/minute). This result was in accordance with several studies^1,24^ which found mean SSFR 0.5 mL/minute in 6-year-old subjects and range 0.3 to 1.5 mL/minute respectively. Results of the present study is in contrast to the study conducted by Sakeenabi and Hiremath.^25^ The difference between the values can be explained by the fact that saliva as a whole is very variable due to its secretion mechanism in which dentition status and developmental changes, such as body weight, height, and maturation of salivary glands, may play a role.^26^

Postdiet counseling values of mean SSFR increased from 0.49 mL/minute to 0.56 mL/minute, which was statistically significant. The postdiet counseling value is similar to the results of Andersson et al^21^ who showed the mean SSFR in 5-year-old healthy subjects to be 0.59 mL/minute. This finding also correlates with the study conducted by Laine et al^1^ who concluded that dietary intervention increased the salivary flow rate in the intervention group as compared with the control group. The increase in the salivary flow may be attributed to greater intake of fiber-rich food items like grains, raw fruits, and vegetables, which require more chewing which in turn is known to increase the salivary flow rate. Also, as per the instructions provided, they might have increased their water uptake.

### Resting pH

In healthy individuals, the resting pH of unstimulated saliva is 6.7 and that of stimulated whole saliva is 6.8 to 7.5.^27^ In the present study, the mean resting pH prior to diet counseling in caries-active individuals was found to be 7.05, which increased to 7.08 postdiet counseling, which was not statistically significant. This finding was in correlation with the study conducted by Dogra et al^28^ and Wu et al,^26^ which stated that caries-active children had a mean resting pH of 7.20 and 7.17 respectively. Preethi et al^7^ evaluated the mean resting pH of saliva in caries-free and caries-active children as 7.18 and 7.15 respectively. They concluded that resting pH had a weak correlation with caries activity. Similar results were seen in a study conducted by Tulunoglu et al.^22^ It has been well documented that the dissolution of enamel occurs when the pH falls below the critical pH, i.e., 5.5, so the values obtained in the study are not adequate to cause deminer-alization of inorganic substance of the tooth.^27^ Hence, it can be speculated that other factors like microflora, diet, and retention of food might have dominated the role of resting pH of saliva to initiate caries.

### Buffering Capacity of Stimulated Saliva

The BCSS neutralizes acids in oral cavity. It plays an important role in the maintenance of salivary pH and remineralization. The buffer capacity of saliva basically depends on three major buffer systems—bicarbonate, phosphate, and protein buffer system.^29^

In the present study, the mean BCSS at baseline was 9.58, which increased to 9.71 postdiet counseling, found to be statistically nonsignificant. This finding is in accordance with several studies^7,22,28^ which concluded that there is a weak correlation between the buffering capacity and caries activity. Our study was in contrast to the study conducted by Ericsson,^30^ which showed that the salivary buffering capacity has a negative relationship with caries incidence.^27^ In the present study, a slight increase in the buffering capacity postdiet counseling may be due to increased salivary flow rate where bicarbonate concentration increases as the flow rate increases, leading to increased buffering capacity.^1^

### Diet Counseling

Measures for diet modification were recommended to all participants of the study. These measures were adopted from the guidelines given by Decker and Loveren.^13^ Patients were instructed (verbally and written instructions were given) to eat balanced diet rich in whole grains, fruit, and vegetables (food items that require more chewing which in turn increases the salivary flow rate) and dairy products like paneer, cheese, curd, milk, etc., to maximize oral and systemic health and reduce caries risk. Emphasis was given on decreasing the eating frequency to reduce repeated exposure to sugars, other fermentable carbohydrates, and acidic beverages, and increasing daily water intake.

### Oratest—Chair-side Caries Activity Test

In the present study, the baseline reading of Oratest ranged from 40 to 130 minutes with a mean of 82.78 minutes. Postdiet counseling reading of Oratest ranged from 70 to 130 minutes with a mean of 99.67 minutes, which was statistically significant. There was an increase in the Oratest reading, suggesting a decrease in the caries activity postdiet counseling. These values were in accordance with the hypothesis given by Rosenberg et al^8^ that higher the infection, lesser was the time taken for the change in the color of the expectorate, reflecting higher oral microbial levels. The reason for the increase in Oratest readings apart from diet counseling can also be due to the sealing of carious lesions within the study period, thus limiting the number of microorganisms present in active carious lesions.

Less time was taken for the initiation of color change in high-caries-risk individuals, and thus the applicability of this test in these patients with rampant caries is suggested for monitoring the preventive measures. This is in accordance with the findings of the following studies: Patalay et al,^31^ Bhasin et al,^10^ Arora et al,^32^ and Saxena et al.^33^

Note that apart from fermentable sugars and acid intake, studies showing association between nutrition and caries are scarce; therefore, further long-term studies are needed to determine the effect of diet on oral health, salivary flow, and salivary composition.

## SUMMARY AND CONCLUSION

- Diet counseling, parental education, and regular motivation can alter salivary parameters, such as USFR and SSFR, which in turn play a major role in caries progression.
- Oratest is a simple, noninvasive, and economic caries activity test, which serves as an index for the success of therapeutic measures and also helps to motivate and monitor the effectiveness of educational programs relating to dietary and oral hygiene procedures. Thus, it can be routinely used to identify high-risk individuals and to motivate them.
